# Clinical utility of hepatitis C virus core antigen assay in the monitoring of direct-acting antivirals for chronic hepatitis C

**DOI:** 10.1371/journal.pone.0229994

**Published:** 2020-03-03

**Authors:** Sheng Feng Lin, Shui-Yi Tung, Kuo-Liang Wei, Chien-Hung Chen, Tsung-Hui Hu, Chien Heng Shen, Te-Sheng Chang, Wei-Ming Chen, Chih-Wei Yen, Jing-Houng Wang, Chao-Hung Hung, Sheng-Nan Lu

**Affiliations:** 1 Division of Hepatogastroenterology, Department of Internal Medicine, Chiayi Chang Gung Memorial Hospital, Chiayi, Taiwan; 2 Division of Hepatogastroenterology, Department of Internal Medicine, Kaohsiung Chang Gung Memorial Hospital and Chang Gung University College of Medicine, Kaohsiung, Taiwan; National Taiwan University Hospital, TAIWAN

## Abstract

**Background:**

Hepatitis C virus core antigen (HCV Ag) assay has been proposed as a more economical alternative to HCV RNA detection. This study aimed to investigate the clinical utility of HCV Ag assay in the monitoring of direct-acting antivirals (DAAs) for chronic hepatitis C patients.

**Methods:**

We analyzed serum samples from 110 patients treated with paritaprevir/ritonavir, ombitasvir, and dasabuvir (PrOD) with or without ribavirin. The levels for both HCV Ag and HCV RNA assessed by COBAS TaqMan HCV (CTM) Test or Abbott RealTime HCV (ART) assay were evaluated at baseline, week 2, 4, and 12 during treatment and 12 weeks after completion.

**Results:**

Baseline HCV Ag levels showed good correlations with HCV viral load (r = 0.879; *p*<0.001); whereas the correlation was slightly stronger with CTM test than with ART assay (*p* = 0.074). The concordance of HCV Ag and HCV RNA undetectability was significantly better in CTM test than in ART assay at week 2 (*p* = 0.003) and week 4 (*p* = 0.003). A sustained viral response 12 weeks off therapy (SVR_12_) was achieved in 108 patients (98%); the HCV Ag assay identified 99% of these patients. Both undetectability of serum HCV Ag and HCV RNA had high positive predictive value at week 2 (98% vs. 100%) and at week 4 (97% vs. 99%) in predicting SVR_12_.

**Conclusions:**

HCV Ag assay may be a feasible alternative to HCV RNA for the determination of SVR_12_ in patients treated with DAAs.

## Introduction

Hepatitis C virus (HCV) infection is one of the major public health problems affecting an estimated 71 million people worldwide [1]. It leads to progressive liver disease including cirrhosis and hepatocellular carcinoma (HCC) in around one-third of the patients in its clinical course [2]. This indicates that HCV eradication is utmost crucial in the prevention of disease progression. With the introduction of direct acting antiviral (DAA) agents, the eradication rate of HCV has been dramatically increasing. DAA has been recommended by the most leading treatment guidelines worldwide based on its less adverse effects, better tolerance, and higher sustained virological response (SVR) rate [3,4].

In the era of interferon-based therapy, measurement of hepatitis C viral load by sensitive molecular techniques is the gold standard for treatment monitoring. Despite the advances in DAA therapy, monitoring of HCV RNA at specific time points before, during, and after therapy is still recommended to understand the treatment adherence and efficacy. However, the current strategy has proven to be rather costly, thus additional methods for screening and monitoring should be necessary. Hepatitis C core Ag (HCV Ag), which forms the internal capsid and is highly conservative and antigenic, has emerged as a marker of HCV infection and an indirect marker of HCV replication [5–7]. The assays for HCV Ag developed in the last decade have been shown to have good correlation with HCV RNA viral load [8,9]. Furthermore, HCV Ag assay may be superior to the current two-step diagnostic approach in terms of time and cost saving benefits. The novel approach has also established its clinical utility in the screening of active HCV infections amongst anti-HCV antibody positive individuals [10].

In routine clinical practice, fully-automated molecular assays for HCV load such as the COBAS TaqMan HCV (CTM) test and the Abbott RealTime (ART) HCV assay are widely used. The limit of detection (LODs) for these assays are 15 international units (IU)/mL and 12 IU/mL, respectively [11–13]. Depending on the assay used, the correlation of serum HCV Ag level with serum HCV load may differ significantly. These differences between assays may have implications for response prediction.

In this study, we attempted to evaluate the clinical utility of serum HCV Ag levels in the monitoring and assessing the treatment response in chronic hepatitis C patients treated with DAA. Furthermore, we compared the concordance of serum HCV core Ag level with HCV RNA levels between these two HCV RNA assays before, during and after DAA therapy.

## Material and methods

### Patients and treatments

From July 2017 to September 2017, a total of 110 patients (63 men and 47 women) treated with 12 weeks of paritaprevir/ritonavir, ombitasvir, and dasabuvir (PrOD) with or without ribavirin were retrospectively enrolled from two hospitals in Taiwan (Chiayi Chang Gung Memorial Hospital and Kaohsiung Chang Gung Memorial Hospital) [14]. These patients had positive anti-HCV antibody and detectable HCV RNA in the serum for more than 6 months prior to the treatment. The severity of fibrosis was ascertained by ultrasonography or fibrosis index based on 4 factors (FIB-4) test. Patients with active HCC (defined as the presence of viable tumors in dynamic images according to the American Association for the Study of the Liver Diseases guidelines [15] or limited life expectancy were excluded. Written informed consents for the use of stored remaining specimens were obtained from all patients prior to the participation in the study. This study was approved by the Research Ethics Committee of Chang Gung Memorial Hospital and was conducted in accordance with the principles of Declaration of Helsinki and the International Conference on Harmonization for Good Clinical Practice.

### Virological measurements

Serum HCV RNA levels were assessed at baseline, week 2, week 4, the end of the treatment, and 12 weeks off therapy. An undetectable HCV RNA level at 12 weeks off therapy was defined as SVR_12_. Serum HCV RNA levels were measured by Abbott RealTime HCV assay (ART; Abbott Molecular, Des Plaines, IL) at Chiayi Chang Gung Memorial Hospital, or CTM, COBAS TaqMan HCV Test (TaqMan HCV; Roche Molecular Systems Inc., Branchburg, N.J.) at Kaohsiung Chang Gung Memorial Hospital. Genotyping of HCV was performed by reverse hybridization assay (Inno-LiPA^TM^ HCV II; Innogenetics N.V., Gent, Belgium) using the HCV-Amplicor products, or RealTime Genotyping II RUO assay (Abbott Molecular, Des Plaines, IL). Serum HCV Ag quantification was retrospectively measured using Abbott ARCHITECT HCV Ag Assay (Abbott Germany, Wiesbaden, Germany) in stored serum samples obtained at the same time points as the HCV RNA. The cut-off value for HCV Ag detection was 3.0 fmol/L; levels below 3.0 fmol/L were considered non-reactive.

### Statistical methods

In this study, the statistical software “SPSS 15.0” was used for data analysis. Continuous data were presented as mean ± standard deviation, and were compared by Student’s t-test or Mann–Whitney *U* test where appropriate. Categorical data were shown as number (percentage) and were compared by chi-square test or Fisher exact test. Linear regression analysis was used to assess the association between HCV Ag and HCV RNA levels. The diagnostic performance of HCV Ag and HCV RNA in the prediction of SVR_12_ was expressed as sensitivity, specificity, positive predictive value (PPV), negative predictive value (NPV), and area under the receiver operating characteristic (AUROC) curve. All statistical tests were 2-tailed, and a *p*-value of less than 0.05 was declared statistically significant.

## Results

The baseline demographic and virological features of the study population are shown in Table 1. Of them, 106 (96.4%) patients were infected with HCV-1b, whereas 4 (3.6%) were infected with HCV-1a. The majority of the patients were diagnosed as having cirrhosis (68%, n = 75), and a tenth of them had HCC but not active (10%, n = 11).

**Table 1:** Baseline characteristics of 110 enrolled patients.

Variables	Mean ± SD or number (%)
Age (yrs)	63.6±10.4
Male gender, n (%)	63 (57)
Prior IFN, n (%)	64 (58)
HBsAg positive, n (%)	8 (7)
Cirrhosis, n (%)	75 (68)
HCC, n (%)	11 (10)
AST (U/L)	87±52
ALT (U/L)	84±82
Platelet (10^3^/μL)	133±49
FIB-4	4.8±3.2
Genotype 1a/1b	4/106
Serum HCV RNA, log IU/mL	5.9±1.0
Serum HCV Ag, log fmol/L	3.4±0.8

Abbreviation: SD, standard deviation; IFN, interferon; HBsAg, hepatitis B surface antigen; HCC, hepatocellular carcinoma; AST, aspartate aminotransferase; ALT, alanine aminotransferase; FIB-4, fibrosis index based on 4 factors; HCV, hepatitis C virus

**Fig 1:**
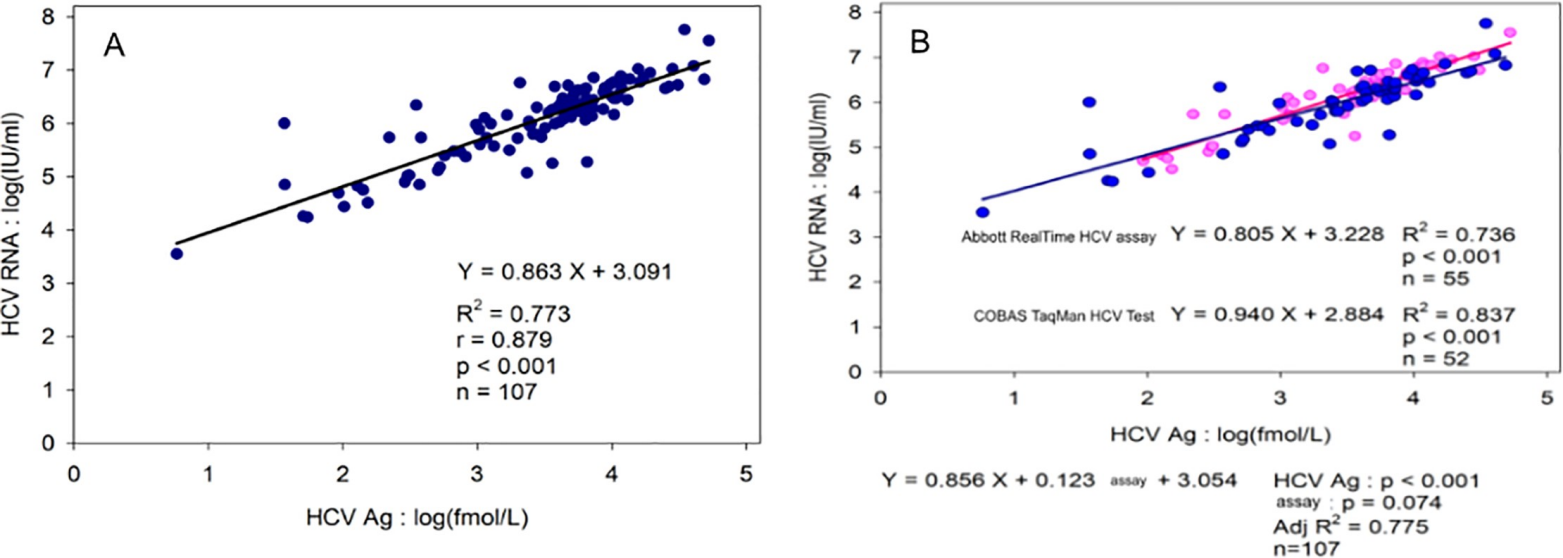
Correlation between baseline HCV core Ag and HCV RNA levels. (A) in total cases (B) in different assays.

Among the patients, 107 (97.3%) were positive for both HCV Ag (>3 fmol/L) and HCV RNA at baseline, while the remaining 3 were positive for HCV RNA only, with low viral load of 124 IU/ml, 876 IU/ml, and 938 IU/ml, respectively. The average of baseline HCV Ag levels was 3.4+/-0.8 log fmol/L. As shown in Fig 1A (Fig 1A), a significant linear correlation between HCV Ag and HCV RNA was found (r = 0.879, *p*<0.001). However, the correlation of HCV Ag levels was slightly stronger with CTM test than with ART assay (*p* = 0.074) (Fig 1B).

**Fig 2:**
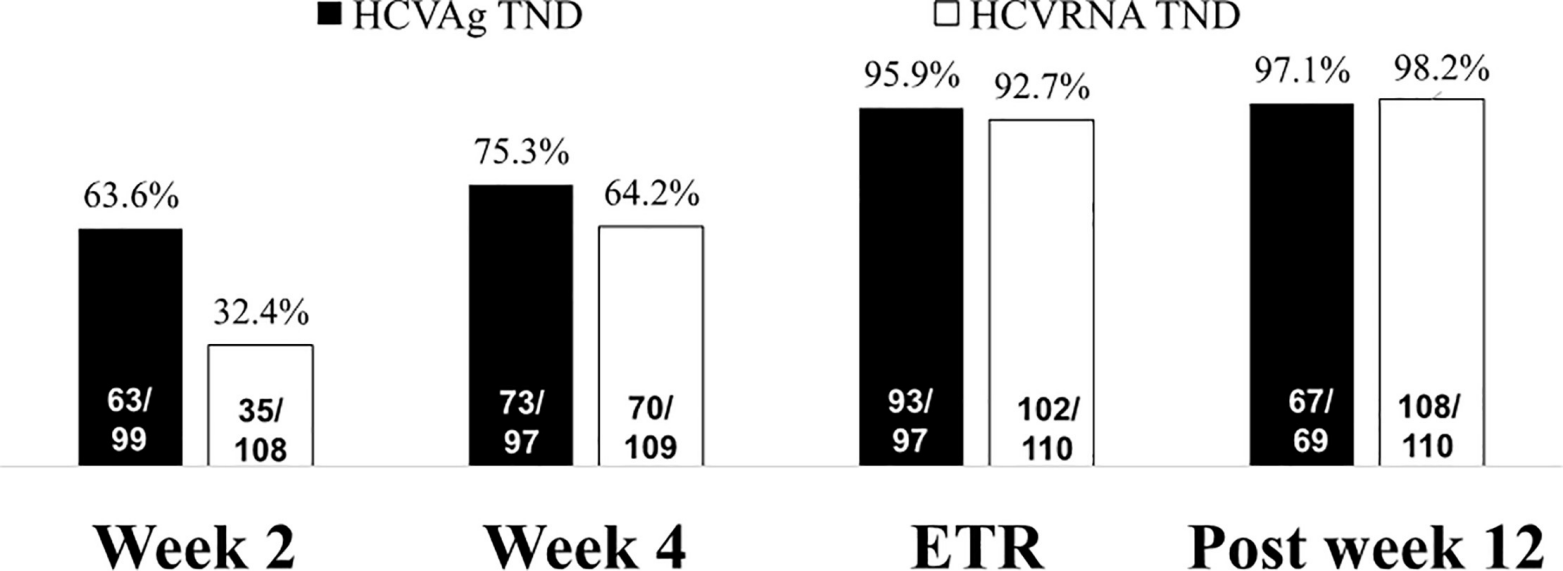
On-treatment virological response of HCV core Ag and HCV RNA during PrOD therapy.

During DAA treatment, serum HCV Ag was undetectable in 64% of patients at week 2, 75% at week 4, and 96% at the end of treatment, while serum HCV RNA became undetectable in 32% of patients at week 2, 64% at week 4, and 93% at the end of treatment, respectively (Fig 2). As regards to different assays, serum HCV RNA became undetectable in 15% of patients at week 2, 39% at week 4, and 82% at the end of treatment by ART assay; and undetectable in 54% of patients at week 2, 91% at week 4, and 100% at the end of treatment by CTM test, respectively. The concordance of HCV Ag and HCV RNA undetectability was significantly better in CTM test than in ART assay at week 2 (*p* = 0.003) and week 4 (*p* = 0.003) (Table 2).

**Table 2:** Concordance of HCV core Ag and HCV RNA undetectability before, during and after DAA therapy.

	HCV RNA TD	HCV RNA TND	Concordance (%)	ART TD	ART TND	Concordance (%)	CTM TD	CTM TND	Concordance (%)	*p-*value
Baseline HCVAg TD	107	0	97.3%	55	0	96.5%	52	0	98.1%	1.000
Baseline HCVAg TND	3	0		2	0		1	0		
W2 HCVAg TD	26	9	54.8%	10	1	36.6%	16	8	69.2%	0.003
W2 HCVAg TND	33	25		25	5		8	20		
W4 HCVAg TD	9	12	63.9	5	1	47.7%	4	11	77.4%	0.003
W4 HCVAg TND	23	53		22	16		1	37		
End-treatment HCVAg TD	1	3	89.7%	1	0	84.1%	0	3	94.3%	0.174
End-treatment HCVAg TND	7	86		7	36		0	50		
Post w12 HCVAg TD	1	1	98.6%	0	0	100%	1	1	98.1%	1.000
Post w12 HCVAg TND	0	67		0	16		0	51		

Abbreviation: HCV, hepatitis C virus; TD, target detected; TND, target not detected; ART, Abbott RealTime HCV assay; CTM, COBAS TaqMan HCV Test. *p*-value: differences between concordance rate of Abbott RealTime HCV assay and COBAS TaqMan HCV Test

At 12 weeks after the treatment, a SVR_12_ was achieved in 108 (98%) patients. Of them, HCV Ag assay identified 99% of these patients, with only one false-positive case with 11.2 fmol/L. The mean serum HCV RNA and HCV Ag levels at baseline was borderline lower in the SVR_12_ patients than those without (5.9 log IU/mL vs. 6.8 log IU/ml, *p* = 0.057) and (3.43 log fmol/L vs. 4.35 log fmol/L, *p* = 0.056) (Table 3).

**Table 3:** Factors associated with SVR_12_.

	SVR_12_ (n = 108)	Non-SVR_12_ (n = 2)	*p*-value
Age (yrs)	65.7±10.4	61.2±7.4	0.509
Male gender, n (%)	62 (57)	1 (50)	1.000
Prior IFN, n (%)	62 (57)	2 (100)	0.509
Cirrhosis, n (%)	73 (68)	2 (100)	1.000
HCC, n (%)	11 (10)	0 (0)	1.000
AST (U/L)	87±52	55±4	0.381
ALT (U/L)	85±82	37±7	0.416
Platelet (10^3^/μL)	133±49	151±27	0.353
FIB-4	4.8±3.2	3.3±1.4	0.502
Genotype 1a/1b	4/104	0/2	1.000
Log HCV RNA (IU/mL)	5.9±0.9	6.8±0.0	0.057
Log HCV Ag (fmol/L)	3.4±0.8	4.4±0.5	0.056

Abbreviation: SVR_12_, sustained virological response12 weeks off therapy; IFN, interferon; HCC, hepatocellular carcinoma; AST, aspartate aminotransferase; ALT, alanine aminotransferase; FIB-4, fibrosis index based on 4 factors; HCV, hepatitis C virus

Table 4 shows the performance of on-treatment HCV Ag and HCV RNA undetectability to predict SVR_12_. At week 2, AUROC for HCV Ag was 0.570 (0.160–0.979), with 64% sensitivity, 50% specificity, 98% PPV, 2.8% NPV, and 64% accuracy. The corresponding AUROC for HCV RNA at week 2 was 0.664 (0.274–1.053), with 33% sensitivity, 100% specificity, 100% PPV, 1.4% NPV and 33% accuracy. At week 4, AUROC for HCV Ag was 0.668 (0.392–0.945), with 75% sensitivity, 0% specificity, 97% PPV, 0% NPV, and 73% accuracy; whereas AUROC for HCV RNA was 0.572 (0.163–0.982), with 64% sensitivity, 50% specificity, 99% PPV, 2.6% NPV and 64% accuracy.

**Table 4:** On-treatment HCV Ag and HCV RNA undetectability to predict SVR12.

On-treatment undetectability	Sensitivity %)	Specificity (%)	PPV (%)	NPV (%)	Accuracy (%)	AUROC (95% CI)
HCV Ag week 2	64	50	98	2.8	64	0.570 (0.160–0.979)
HCV RNA week 2	33	100	100	1.4	33	0.664 (0.274–1.053)
HCV Ag week 4	75	0	97	0	73	0.668 (0.392–0.945)
HCV-RNA week 4	64	50	99	2.6	64	0.572 (0.163–0.982)

Abbreviation: HCV, hepatitis C virus; SVR_12_, sustained virological response12 weeks off therapy; PPV, positive predictive value; NPV, negative predictive value; AUROC, area under Receiver Operating Characteristic

## Discussion

The efficacy of antiviral therapy for HCV is based on the testing for HCV RNA with sensitive techniques. However, the most recent recommendations from prestigious guidelines suggest that the measurement of HCV Ag levels in serum or plasma can be used to as an alternative to HCV RNA level to monitor treatment efficacy when HCV RNA assays are not available or not affordable [3]. In principle, HCV Ag demands less professional training to operate and has a much shorter turnaround time that results in available data within one hour as opposed to nearly seven hours for HCV-RNA. In our study, we showed that HCV Ag and HCV RNA presented similar kinetics in DAA treatment, both during treatment and after follow-up. The quantification of HCV Ag had a high PPV of 98% at week 2 and 97% at week 4 in predicting SVR_12_, which was as high as HCV RNA. Moreover, both undetectability of serum HCV Ag and HCV RNA did not have a high accuracy and AUROC, suggesting that serial measurements during DAA treatment by using either HCV Ag or HCV-RNA had only a limited value in predicting the SVR_12_. [16]

As expected, HCV Ag might be less sensitive and false-negative in cases with very low levels of HCV RNA [17]. In our study, three false-negative patients at baseline had HCV RNA level below 1000 IU/mL. One may go on to conclude that the clinical utility of the HCV Ag may include the screening for potential HCV patients in the community setting. The benefits of the method may not only include point-of-care screening, but also allows for rapidly available results and cost savings. While at 12 weeks after the treatment, the concordance of HCV Ag and HCV RNA levels was extremely high (99%), with only one false-positive case (11.2 fmol/L). Our data suggested that HCV Ag could be used as an alternative endpoint of DAA treatment at 12 weeks off therapy, which was against the recent guideline indicating that HCV Ag should be assessed at 24 weeks after the end of treatment to determine outcome in patients with detectable HCV Ag prior to therapy [3].

Although the viral kinetics of HCV Ag and HCV RNA were similar during DAA therapy, the decline rate somewhat differed between these two tests. In our study, HCV Ag levels declined more rapidly when compared to HCV RNA in the early stages (undetectability of 63.6% vs. 32.4% at week 2 and 75.3% vs. 64.2% at week 4, respectively). These data were comparable with those reported in earlier studies [18,19]. However, of note, the concordance of HCV Ag and HCV RNA undetectability was significantly different when comparing the ART assay and CTM test. Despite no difference at baseline, end of the treatment, and 12 weeks off treatment between the two assays, we provided the first evidence that the concordance of HCV Ag and HCV RNA undetectability was significantly better in CTM test than in ART assay at week 2 (*p* = 0.003) and week 4 (*p* = 0.003). A previous study has demonstrated that increasing the HCV RNA cut-off level to 1000 IU/mL could reduce the number of discordant results of HCV Ag and HCV RNA during DAA treatment [20]. However, no subjects with HCV RNA level more than 1000 IU/mL at week 2 and week 4 were found in our series. Further studies with larger sample size would be required to better comprehend this phenomenon.

Consequent to the retrospective design, the main limitation of this study was that there were missing samples for assessing HCV Ag at various time points. Only samples that were systemically stored in sufficient quality over the entire study period were used. Another limitation was that only genotype 1 patients were enrolled in this study, thus the absence of data from other genotypes limited the reference values. However, in our previous study, there was strong correlation in both genotype 1 and genotype 2 between HCV Ag and HCV RNA [9]. We speculated that our findings could be expended to patients with other genotypes but further studies should be needed to clarify this issue.

## Conclusion

In conclusion, our study found the HCV Ag a feasible alternative to the HCV RNA level for the determination of SVR_12_ in patients treated with DAAs, offering similar clinical information while spending much less time and expenses. However, on-treatment HCV Ag is not a good predictor of achieving SVR12, despite high PPV at week 2 and at week 4. CTM test showed better concordance of HCV Ag and HCV RNA undetectability compared to ART assay during DAA therapy.

## Supporting information

S1 Dataset (SAV)
